# *In situ* label-free imaging for visualizing the biotransformation of a bioactive polyphenol

**DOI:** 10.1038/srep02805

**Published:** 2013-09-30

**Authors:** Yoon Hee Kim, Yoshinori Fujimura, Takatoki Hagihara, Masako Sasaki, Daichi Yukihira, Tatsuhiko Nagao, Daisuke Miura, Shinichi Yamaguchi, Kazunori Saito, Hiroshi Tanaka, Hiroyuki Wariishi, Koji Yamada, Hirofumi Tachibana

**Affiliations:** 1 Faculty of Agriculture, Kyushu University, 6-10-1 Hakozaki, Higashi-ku, Fukuoka 812-8581, Japan; 2 Innovation Center for Medical Redox Navigation, Kyushu University, 3-1-1 Maidashi, Higashi-ku, Fukuoka 812-8582, Japan; 3 MS Business Unit, Life Science Business Department, Analytical and Measuring Instruments Division, Shimadzu Corporation, 1 Nishinokyo Kuwabaracho, Nakagyo-ku, Kyoto 604-8511, Japan; 4 Bruker Daltonics K.K., 3-9 Noriya-cho, Kanagawa-ku, Yokohama 221-0022, Japan; 5 Department of Applied Chemistry, Graduate School of Science and Engineering, Tokyo Institute of Technology, 2-12-1 Ookayama, Meguro, Tokyo 152-8552, Japan; 6 Food Functional Design Research Center, Kyushu University, 6-10-1 Hakozaki, Higashi-ku, Fukuoka 812-8581, Japan; 7 These authors contributed equally to this work.

## Abstract

Although understanding the high-resolution spatial distribution of bioactive small molecules is indispensable for elucidating their biological or pharmacological effects, there has been no analytical technique that can easily detect the naïve molecular localization in mammalian tissues. We herein present a novel *in situ* label-free imaging technique for visualizing bioactive small molecules, using a polyphenol. We established a 1,5-diaminonaphthalene (1,5-DAN)-based matrix-assisted laser desorption/ionization-mass spectrometry imaging (MALDI-MSI) technique for visualizing epigallocatechin-3-*O*-gallate (EGCG), the major bioactive green tea polyphenol, within mammalian tissue micro-regions after oral dosing. Furthermore, the combination of this label-free MALDI-MSI method and a standard-independent metabolite identification method, an isotopic fine structure analysis using ultrahigh-resolution mass spectrometer, allows for the visualization of spatially-resolved biotransformation based on simultaneous mapping of EGCG and its phase II metabolites. Although this approach has limitations of the detection sensitivity, it will overcome the drawbacks associated with conventional molecular imaging techniques, and could contribute to biological discovery.

The representative bioactive small molecule, (–)-epigallocatechin-3-*O*-gallate (EGCG) (Fig. 1a), is the most abundant polyphenol in green tea (*Camellia sinensis* L.). Many studies have revealed that EGCG possesses various pharmacological properties, such as anti-cancer, anti-atherosclerosis, anti-obesity, and neuroprotective effects^1^,^2^,^3^,^4^. To elucidate the precise mechanism underlying the bioactivity of this polyphenol, spatiotemporal information is needed. Although some studies have visualized its tissue distribution by fluorescence imaging, cerium chloride staining, and radioactive labeling assays^5^,^6^,^7^, the spatiotemporal information has been lacking because of the absence of an analytical technique that can easily detect the localization of the naïve polyphenol. Conventional molecular imaging generally requires labeling steps that are time-consuming, expensive, and labor-intensive. In addition, the molecular discriminating powers of these techniques are insufficient for visualizing a target compound and its metabolites simultaneously. A label-free molecular imaging technique could overcome these issues, but the development of such a technique has been a challenge.

Mass spectrometry imaging (MSI) is a new technology capable of determining the naïve distribution of ionizable biological molecules in tissue sections without any labeling on the basis of their specific mass-to-charge ratios. This technique can theoretically detect target molecules and their metabolites simultaneously in a single analysis, and is now widely used for *in situ* imaging of endogenous and exogenous molecules such as proteins, lipids, drugs, and their metabolites^8^,^9^,^10^. It is a potential tool for the pathological analysis and understanding diseases or pharmaceutical mechanisms. Matrix-assisted laser desorption/ionization (MALDI) is a commonly available ionization method used for MSI. MALDI-MSI, using traditional major matrices, such as 2,5-dihydroxy benzoic acid (DHB), sinapinic acid (SA), and α-cyano-4-hydroxycinnamic acid (CHCA), can visualize macromolecules such as lipids and proteins/peptides. However, small molecules are not easily detected by MALDI-MSI because many matrix and/or matrix-analyte cluster ion peaks are observed in the low mass range (*m/z* < 700). In contrast, we recently reported that MALDI-MS with 9-aminoacridine (9-AA) achieved great improvement for the sensitivity of detection of endogenous low-molecular-weight metabolites in the negative ionization mode^11^,^12^,^13^. This technique also visualized both drastic and subtle changes in the spatiotemporal distributions of various cerebral metabolites in response to pathological perturbation. However, it still remains unclear whether such highly sensitive MALDI-MSI techniques can visualize low-molecular-weight bioactive polyphenols. Therefore, in this study, we attempted to establish an *in situ* label-free technique for the simultaneous imaging of a bioactive polyphenol and its metabolites in mammalian tissues following *in vivo* dosing.

## Results

### The determination of the optimum matrix for ionization of EGCG

**Figure 1:**
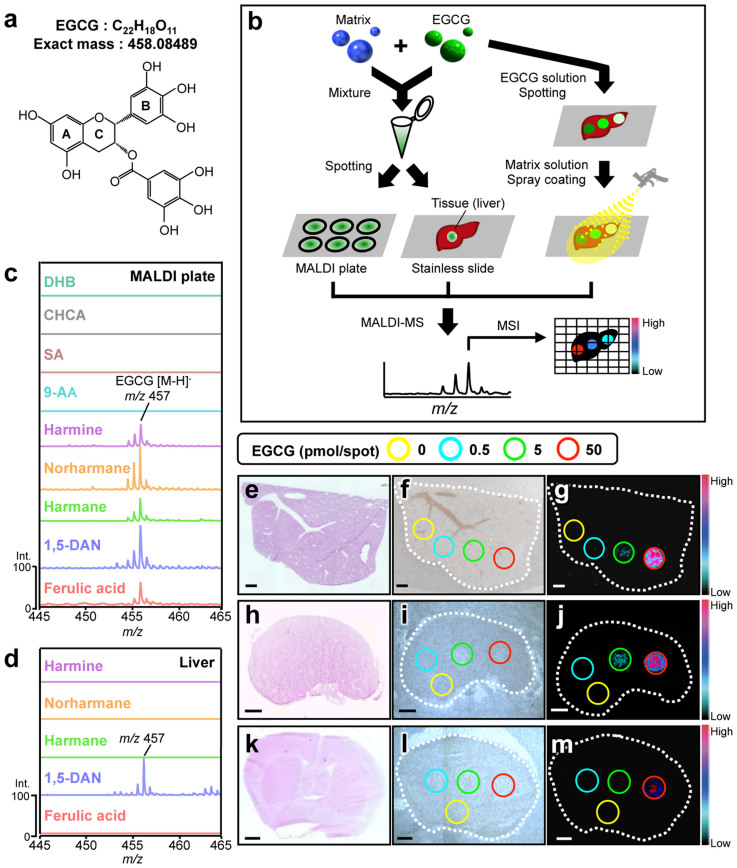
Screening of the optimum matrix for detection of EGCG by MALDI-MS. (a) Chemical structure of EGCG. (b) A schematic representation of matrix screening. Mass spectral observations for each EGCG-matrix candidate mixture at *m/z* 457 [EGCG (C_22_H_18_O_11_)–H^+^]^−^ by MALDI-MS analysis on (c) the stainless steel MALDI plate or (d) the mouse liver section in negative ionization mode. Each data was represented as the relative signal intensity with the intensity of the strongest intensity peak as 100%. MALDI-MSI of EGCG spotted on three representative mouse tissue sections was performed. Three different images of (e, h, k) H&E staining, (f, i, l) optical microscopy, and (g, j, m) MALDI-MS at *m/z* 457 in mouse (e–g) liver, (h–j) kidney, and (k–m) brain sections are shown. A series of EGCG solutions were spotted on each tissue section. MSI data were acquired with 50 μm spatial resolution with 10 shots/data point. Scale bar = 1.0 mm.

To effectively ionize the analyte in MALDI-MS, the optimum matrix needs to be determined. Because little is known about what matrices can ionize polyphenols, we screened 41 chemicals as potential matrices for the green tea polyphenol EGCG (Fig. S1). A solution of each chemical (10 mg/mL in methanol (MeOH)) was mixed with an equal volume of an EGCG solution, and the mixture was spotted onto a stainless steel MALDI plate (50 pmol EGCG/spot) and analyzed by MALDI-TOF-MS (Fig. 1b). EGCG peaks were not observed with DHB, CHCA, SA, or 9-AA, which are the most effective major matrices for ionizing small molecules^14^. However, 1,5-diaminonaphthalene (1,5-DAN), harmane, norharmane, harmine, and ferulic acid all allowed for the detection of EGCG (*m/z* 457 [M–H]^−^) in negative ionization mode without any background peaks (Fig. 1c, Table. S1 and Fig. S2a). This detection was strongly dependent on the solvent used to prepare the mixture solution (Fig. S2b). Because ion suppressive effects are predominantly caused by competing endogenous species in tissues^14^, we determined which of these matrix candidates in its corresponding optimum solvent (1,5-DAN or ferulic acid in acetone (ACE), or harmane, norharmane, or harmine in MeOH) could effectively ionize EGCG spotted on the tissue sections (Fig. 1b). Only 1,5-DAN could ionize EGCG on the liver section without producing any background peaks (Fig. 1d and Fig. S2c).

Therefore, we used 1,5-DAN to attempt two-dimensional visualization of EGCG spotted on normal mouse tissue sections (Figs. 1e–m). Because the different structural and biological compositions of different tissues may influence the ionization efficiency of EGCG, we used three representative tissue sections (liver, kidney, and brain) for MALDI-MSI. After spotting EGCG on these tissue sections, 1,5-DAN was applied by spray coating (Fig. 1b). An ion image of *m/z* 457 [M–H]^−^ was obtained at more than 5 pmol in the liver and kidney sections, but only a level of 50 pmol was found in the brain section (Figs. 1g, j, m), thus indicating that 1,5-DAN detects EGCG in a manner that depends on the tissue matrix. Although EGCG does not have a leaving group (Fig. 1a) for effective ionization, as previously reported in major available matrices^14^, we found that 1,5-DAN could effectively ionize EGCG on all tissue sections.

### *In vivo* administered EGCG can be visualized by 1,5-DAN-based MALDI-MSI in mouse liver sections

**Figure 2:**
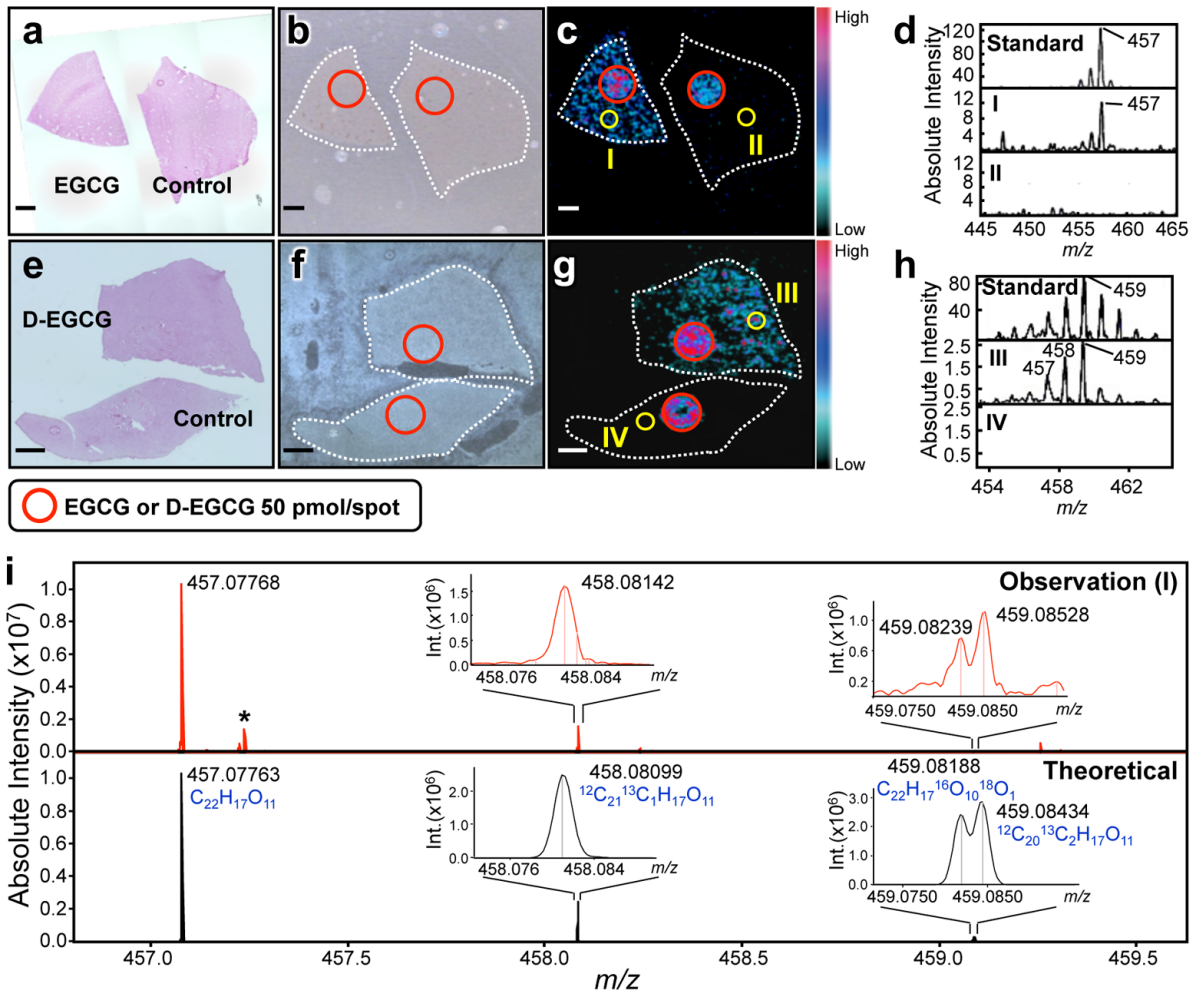
MALDI-MSI of orally administrated EGCG in mouse liver sections. (a–d) MSI of EGCG in mouse liver section. Three different images of (a) H&E staining, (b) optical microscopy, and (c) MALDI-TOF-MS at *m/z* 457 are shown. An additional EGCG spot (red circle) was visualized as the positive and internal control. MSI data were acquired with 50 μm spatial resolution with 10 shots/data point. Scale bar = 1.0 mm. Mass spectral observations of (d) *m/z* 457 were obtained in the region of interest (I & II) indicated in panel c. To exclude the possibility that the EGCG peak observed on the tissue section is endogenous, the same experiment was performed in deuterated EGCG (D-EGCG)-administrated mouse (e–h). The main peak of D-EGCG was observed at *m/z* 459 (Fig. S6). (i) For mass spectral identification of the EGCG peak, the isotopic fine structure of EGCG peak within the region of interest (I) indicated in panel c was measured using ultrahigh-resolution MALDI-FT-ICR-MS. Theoretical peaks of EGCG (C_22_H_17_O_11_) were shown in negative ionization mode, and isotopic peaks were observed in the (M–H^+^ + 1)^−^ and (M–H^+^ + 2)^−^ regions. These peaks were theoretically assigned to the substitution of a stable isotope for each element. Asterisk shows background peaks.

We next applied this label-free technique, 1,5-DAN-based MALDI-MSI, to visualize EGCG in mouse tissues 1 h after the oral administration of EGCG. At this time, corresponding to the peak accumulated time^2^, the accumulation of EGCG was observed in the liver (149.55 ± 37.51 nmol/g) and kidney (11.17 ± 1.82 nmol/g) tissues by liquid chromatography-MS (LC-MS) (Figs. S3a–d), but not in brain tissue. Therefore, MSI was performed in liver and kidney tissues. After spotting EGCG (50 pmol) on tissue section as a positive control, 1,5-DAN was applied by spray coating and the samples were subjected to MALDI-TOF-MS (Figs. 2a–d). An ion image of *m/z* 457 was obtained in the EGCG-administered liver section, while this peak was not observed in the control section (Figs. 2c, d).

To confirm that the peak was not endogenous, we synthesized deuterated EGCG (D-EGCG) from the intact EGCG (Figs. S4, S5) and used it for the dosing and subsequent MSI (Figs. 2e–h). Strong deprotonated peaks at *m/z* 458 and 459 (intensity: 459 > 458) were detected by MALDI-MS, which reflected the deuteration rate of 66% D at the 2′ and 6′ position in the B ring (Figs. S6a, b). One hour after the administration of D-EGCG, it was observed in the liver (Fig. S6c) and *m/z* 459 was the main peak (Figs. S6d–f). In MALDI-MSI, the ion image and peak at *m/z* 459 were observed in the D-EGCG-administered group, but not in the control group (Figs. 2g, h). Furthermore, the 457/459 peak ratio of the liver sample was almost identical to that of the standard (Fig. 2h). These results indicate that the *m/z* 457 peak shown in Fig. 2c is not endogenous.

To further establish that the *m/z* 457 peak is the dosed EGCG, we performed an isotopic fine structure analysis using ultrahigh-resolution Fourier-transform ion cyclotron resonance (FT-ICR)-MS. Unlike conventional MALDI-TOF-MS, FT-ICR-MS has the highest mass resolving power of all current MS instrumentation (>100,000) as well as sub ppm mass accuracy, and can unambiguously determine one elemental composition in a standard-independent manner, as we reported previously^15^. In region (I) of Fig. 2c, the deprotonated ion peak [M–H]^−^ was observed at *m/z* 457.07768 (Fig. 2i, Observation (I)). Two isotopic peaks were also observed around *m/z* 458.08 and 459.08. MALDI-FT-ICR-MS could clearly separate several isotopic peaks caused by isotopic substitution, and the observed peaks were almost identical to the theoretical peaks of EGCG (Fig. 2i, Theoretical), demonstrating that *m/z* 457 was indeed the dosed EGCG. Taken together, these findings indicated that we succeeded in acquiring two-dimensional images of the green tea polyphenol, EGCG, in the tissue sections using a newly developed label-free imaging technique, 1,5-DAN-based MALDI-TOF-MSI.

### Simultaneous visualization of orally administered EGCG and its metabolites in tissue micro-regions

**Figure 3:**
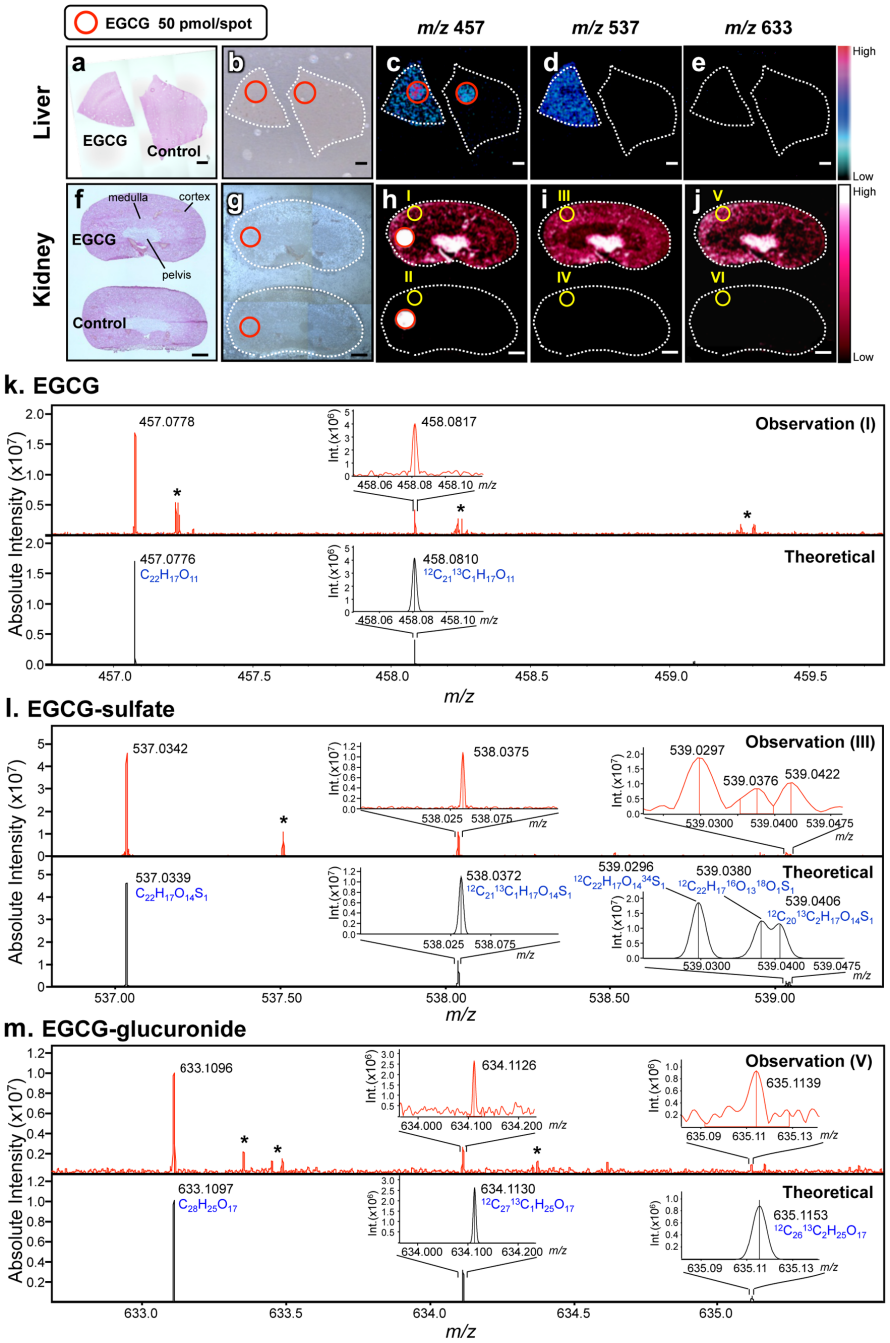
Visualization of dosed EGCG and its phase II metabolites in tissue micro-regions. (a–j) Simultaneous visualization of EGCG and its phase II metabolites in (a–e) liver and (f–j) kidney sections. Three different images are shown in (a, f) H&E staining, (b, g) optical microscopy, and MALDI-TOF-MS: (c, h) EGCG (*m/z* 457), (d, i) EGCG-sulfate (*m/z* 537), and (e, j) EGCG-glucuronide (*m/z* 633). An additional EGCG spot (red circle) was visualized as the positive and internal control. (k–m) For mass spectral identification, the isotopic fine structures were measured by MALDI-FT-ICR-MS in the region of interest (I, III, and V) indicated in panels h–j. Theoretical peaks of (k) EGCG (C_22_H_17_O_11_), (l) EGCG-sulfate (C_22_H_17_O_14_S_1_), and (m) EGCG-glucuronide (C_28_H_25_O_17_) were shown in negative ionization mode, and isotopic peaks were observed in the (M–H^+^ + 1)^−^ and (M–H^+^ + 2)^−^ regions. These peaks were theoretically assigned to the substitution of a stable isotope for each element. Asterisk shows background peaks.

**Figure 4:**
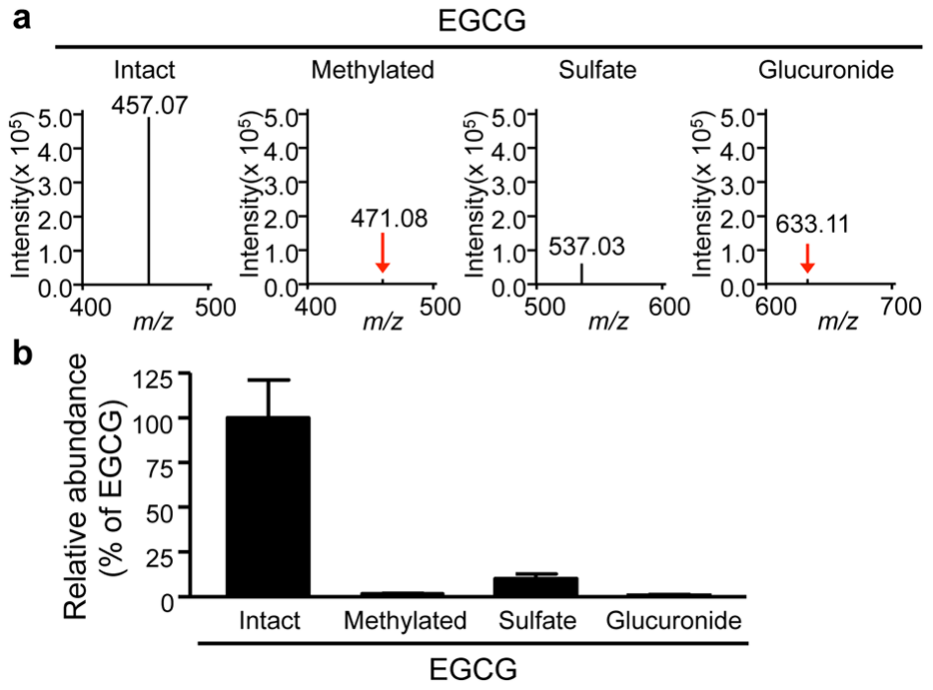
The relative abundance of EGCG and its phase II metabolites from kidney tissue extracts. (a) The whole tissue extracts of kidney from EGCG-dosed mouse were subjected to LC-MS measurement. Mass spectra at *m/z* 457 (EGCG), 471 (methylated EGCG), 537 (EGCG-sulfate), and 633 (EGCG-glucuronide) are shown. (b) The relative peak abundance of such EGCG phase II metabolites to EGCG is represented as the mean ± S.D. of six mice.

Understanding the biotransformation (i.e., metabolism) of *in vivo* administered bioactive polyphenols is indispensable for elucidating their precise mechanism(s) of action^2^,^16^. However, the high-resolution spatial distribution (i.e., localization at the tissue micro-region level) of polyphenols remains largely unclear. In the present study, we attempted to simultaneously visualize EGCG and its metabolites in liver and kidney sections (Figs. 3a–j). In the liver, an ion peak at *m/z* 537 was detected in the EGCG-administered group, but not in the control group (Fig. 3d and Fig. S7d). This peak was also observed in LC-MS of the liver extract (Figs. S7e–g), and a product ion peak at *m/z* 457, corresponding to EGCG [C_22_H_18_O_11_–H^+^]^−^, was detected by MS/MS of *m/z* 537. Subtraction of these peaks, yielding a value of 80 Da, suggested that *m/z* 537 might be the deprotonated peak of EGCG-sulfate [C_22_H_18_O_14_S_1_–H^+^]^−^. Although this metabolite standard was not commercially available, the isotopic fine structure analysis using MALDI-FT-ICR-MS showed that *m/z* 537 was identical to the theoretical peak of EGCG-sulfate (Fig. S7h). One of the phase II biotransformation products of EGCG^16^, EGCG-sulfate, was homogeneously detected in the liver, and its distribution pattern was similar to that of EGCG (Figs. 3c, d). The distribution of EGCG and its sulfate was also observed in the kidney (EGCG: Fig. 3h and Fig. S8e; EGCG-sulfate: Fig. 3i and Figs. S8f, h–j). Furthermore, unlike liver (Fig. 3e), an ion peak at *m/z* 633, corresponding to EGCG-glucuronide [C_28_H_26_O_17_–H^+^]^−^, was observed in kidney (Fig. 3j and Figs. S8g, h–j). A series of polyphenols were assigned by MALDI-FT-ICR-MS in a standard-independent manner (Figs. 3k–m). Interestingly, MALDI-TOF-MSI revealed for the first time that the localization patterns in kidney compartments (pelvis, medulla, and cortex) were clearly different among EGCG and its phase II metabolites (Figs. 3h–j). The highest abundance of the three EGCG derivatives was observed in the pelvis, the funnel-like dilated proximal part of the ureter in the kidney. In the phase II metabolism, EGCG undergoes predominantly methylation, sulfation, and glucuronidation^16^. Such biotransformation products were observed in whole tissue extracts of kidney by LC-MS analysis (Fig. 4), but major methylated form (*m/z* 471 [M–H]^−^) was not detected on kidney tissue sections by MALDI-MS (Fig. S9). In addition to the experimental dosage of EGCG (2,000 mg/kg), we performed MALDI-MSI test using mouse tissue sections after oral dosing at a normal intake level (20 mg/kg). As shown in Fig. S10, EGCG and its phase II metabolites were not visualized in both kidney and liver tissue sections. These results suggested limitations of the method related to the detection sensitivity.

**Figure 5:**
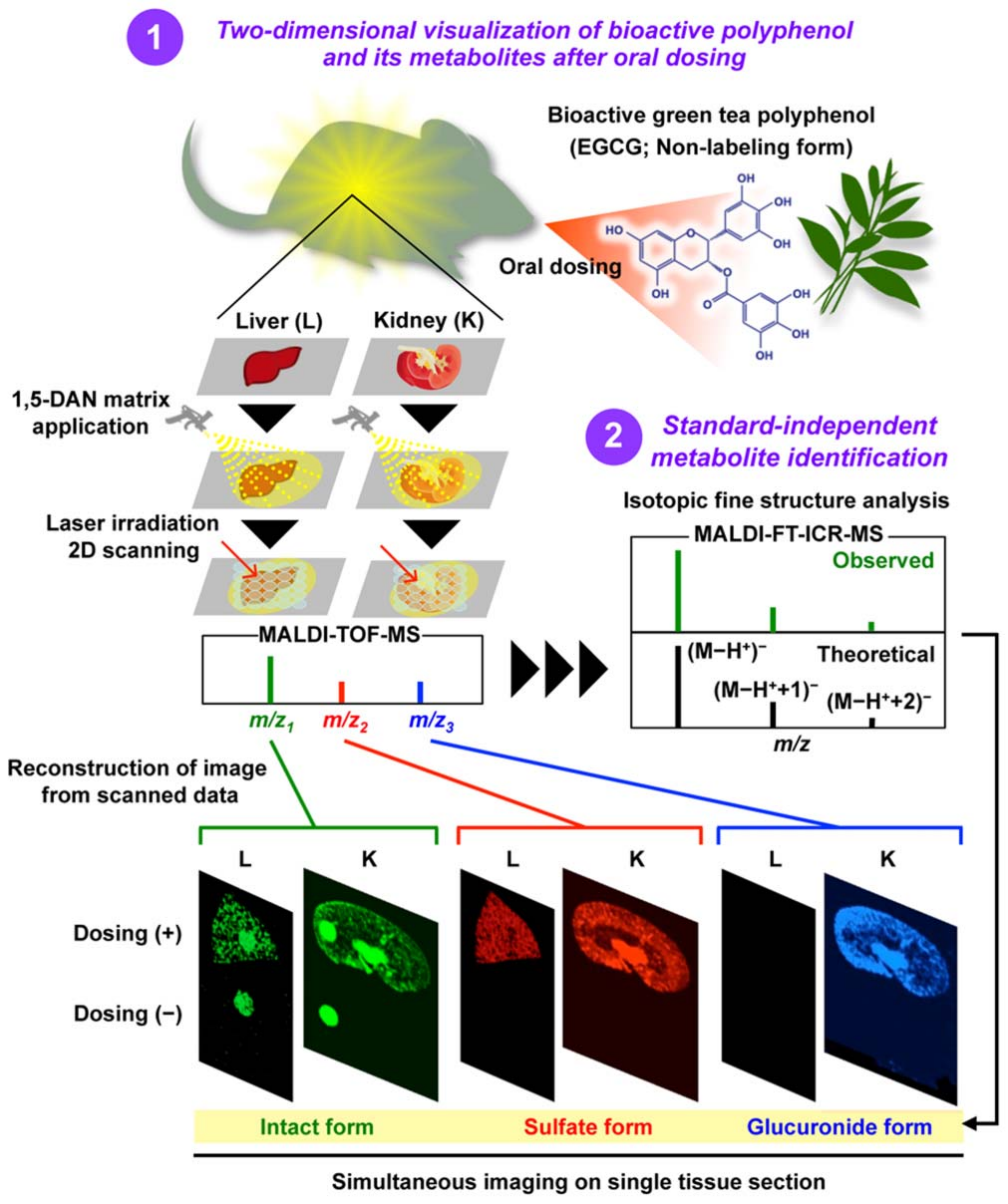
*In situ* label-free imaging system combing MALDI-MSI technique with standard-independent metabolite identification method.

At least, our proposed *in situ* label-free imaging approach (Fig. 5), combing MALDI-MSI and the isotopic fine structure analysis, enabled us to visualize an orally administered bioactive polyphenol and its metabolites simultaneously in tissue micro-regions. These findings will be useful for better understanding the metabolism of EGCG and other polyphenols.

## Discussion

In this study, we demonstrated for the first time that MALDI-MSI could be used to visualize an orally administered green tea polyphenol, EGCG, and its metabolites simultaneously in mammalian tissues without any labeling. Conventional label-based molecular imaging is time-consuming, expensive, and labor-intensive. In addition, traditional MS platforms (LC-MS and GC-MS) suffer from the lack of both spatial information (i.e., average information in tissue extracts) and the ability to resolve parent molecule from its metabolites. Our new label-free imaging technique was able to overcome such drawbacks.

For MALDI-MSI, the matrix selection is one of the most important issues to ensure the highly sensitive imaging of the target compound. Many distribution studies of endogenous metabolites and drugs have proposed optimum matrices, such as DHB, CHCA, SA, and 9-AA^13^,^14^, but there has been no information about a matrix capable of detecting trace amounts (i.e., physiologically relevant concentrations) of polyphenolic compounds. To our knowledge, the present study was the first to screen various matrices to identify the optimum matrix for ionizing a bioactive polyphenol and to visualize its distribution in mammalian tissues. The 1,5-DAN, the optimal matrix for EGCG, has been reported to enable the detection of lipids and peptides/proteins in positive or negative ionization mode^17^,^18^. Recent studies have proposed the deprotonation^19^ and electron transfer reactions^20^ as the potential mechanisms of the negative-mode ionization of an analyte, but their relationship to the ability of 1,5-DAN to ionize EGCG is unclear, and further studies will require to elucidate how 1,5-DAN helps to enable the ionization of EGCG.

Generally, library or database search strategies using the exact MS and MS/MS pattern of mass spectra of known and available compounds are well-established chemical annotations with MS^21^. This strategy is not a data-driven method, but is completely dependent on the spectral databases or comparisons with reference authentic standard spectra, despite the fact that commercially available compounds are within 20% of whole biological metabolites^22^. The elemental composition represents one of the most important pieces of information for the determination of the structure of completely unknown metabolites. We recently found that ultrahigh-resolution FT-ICR-MS could determine the elemental composition of low-molecular-weight metabolites on the basis of isotopic peak ratios, without the requirement of MS/MS^15^. Quantitatively detected isotopic peaks of elements (C, H, O, N, P, and S) are closely matched to the natural abundance of each element. These data successfully led us to unambiguously determine the one elemental composition in a standard-independent manner. On the other hand, conclusive identification of a target compound and its metabolites in tissues often becomes problematic due to the presence of many interfering peaks from endogenous species and matrix within the low mass range. TOF-MS in particular suffers from low mass resolution that is frequently insufficient to resolve compound peaks from endogenous species. However, this issue was not observed in our TOF-MS analysis using 1,5-DAN (Figs. 2d and h, Fig. S7d, and Figs. S8e–g). In addition, our data (Figs. 2i and 3k–m) showed that the ultrahigh-resolution FT-ICR-MS analysis could resolve peaks of orally administered EGCG or its metabolites from matrix and endogenous peaks. Furthermore, the FT-ICR-MS data (Figs. 2i and 3k–m) and its complementary LC-MS data (Figs. S3d, 7g, and 8j) suggest that even low abundance compounds (e.g., a bioactive polyphenol with poor bioavailability) can be sensitively identified without the requirement for direct MS/MS of peaks detected on the tissue section. Therefore, the combination of conventional TOF-MSI and isotopic fine structure analysis using ultrahigh-resolution FT-ICR-MS may become an effective strategy for analyzing the spatially-resolved metabolism of bioactive polyphenols.

Understanding the metabolic fates of bioactive polyphenols is indispensable for determining their *in vivo* molecular mechanisms^2^. Some studies have reported that green tea polyphenols are subjected to phase II biotransformation and undergo predominantly methylation, glucuronidation, and sulfation in the intestine, liver, and kidneys^16^. However, both the functions of the metabolites and their localizations in different tissue micro-regions were unclear^23^. In LC-MS experiments, EGCG and its major conjugates (methylated, sulfated, and glucuronidated forms) were observed in the kidney tissue extract (Figs. 4a, b). The peak abundance of such conjugates was markedly lower than that of EGCG. Nevertheless, both sulfated and glucuronidated forms were detected in reproducible MALDI-MSI measurements, but there was no peak of methylated form (*m/z* 471 [M–H]^−^, Figs. S9b–g). In negative ionization mode MALDI-MS, it was known that phosphorylated compounds and carboxylic acids were efficiently ionized, indicating that compounds with leaving groups, including phosphate and carboxylic groups, readily undergo the deprotonation^11^,^12^,^13^. Unlike methylation, sulfation or glucuronidation can introduce leaving group (sulfate or carboxylic group, respectively) into EGCG. Therefore, in negative ionization mode MALDI-MS using 1,5-DAN, the introduction of such an ionizable group may contribute to preferable ionization, higher MALDI efficiency, of EGCG phase II conjugates, in spite of their lowered tissue abundance compared to EGCG. Although the bioavailability of EGCG is very low^2^,^16^, we were first able to visualize the distribution of EGCG phase II metabolites in liver and kidney sections after oral dosing (Fig. 3). This discovery raises several issues for further investigations, such as the elucidation of the potential relationship between the precise locations where EGCG can directly exhibit its bioactivity and the region-specific active form.

Although the present MALDI-MSI has attractive advantages such as label-free imaging and simultaneous detection of an orally dosed polyphenol and its metabolites, the technique could not visualize EGCG and its phase II metabolites in the range of normal oral dosages of EGCG (20 mg/kg, Fig. S10). For practical use of our proposed 1,5-DAN-MALDI-MSI technique, further improvement of the detection sensitivity, especially improvement of MALDI efficiency based on matrix development, is required. In addition, the temporal analysis, pathological models, and visualization of other metabolites will be required to unravel both the biological consequence of biotransformation of the green tea polyphenol and its mechanism(s) of action.

Our new label-free imaging technique, using a combination of 1,5-DAN-based MALDI-MSI and a standard-independent metabolite identification method (Fig. 5), will open new avenues for investigating the spatiotemporal behavior of a bioactive polyphenol, and could be broadly applicable to emerging issues in the absorption and metabolism of various bioactive small molecules, such as phytochemicals and drugs.

## Methods

### Materials

All chemicals used were of analytical reagent grade. Matrix chemicals and solvents were purchased from Wako Pure Chemical Industries, Ltd or Sigma-Aldrich if not stated otherwise. Ferulic acid and THAP were obtained from Fluka, and DHB was purchased from TCI MARK. EGCG was purchased from Sigma-Aldrich.

### Animal

Male 6-week-old C57BL/6J mice were purchased from Kyudo Co., LTD, and were housed in a temperature- and humidity-controlled room, and fed a commercial diet and water *ad libitum*. This experiment was carried out according to the guidelines for animal experiments at the Faculty of Agriculture, Kyushu University. The study protocol was approved by the Animal Care and Use Committee of Kyushu University, Fukuoka, Japan. The approval number for the animal experiment is A24-052-1.

### Detection of EGCG spotted on a stainless steel MALDI sample plate

EGCG was dissolved in distilled water at 5 mM stock concentration and stored at −20°C until assay. The stock solution was diluted with 100% MeOH, ACE or ACN to 100 μM to prepare the analyte solutions. Forty-one chemicals were initially screened as matrices for the detection of EGCG with MALDI-TOF-MS (AXIMA Performance, Shimadzu). Ten microliters of 100 μM EGCG (100% MeOH) was added to an equal volume of one of the matrix solutions (10 mg/mL in 100% MeOH). Aliquots (1 μL) of the matrix-EGCG mixtures were spotted onto a 384-well stainless steel MALDI sample plate (50 pmol EGCG/well, on-plate, *n* = 5) and analyzed in both negative and positive ionization modes. The instrument was equipped with a 337 nm N_2_ laser. Each sample spot was analyzed for 242 shots, and all spectrometric data were processed and analyzed using Shimadzu Biotech Launchpad software. Among the 41 chemicals tested, we selected five matrix candidates (ferulic acid, 1,5-DAN, harmane, norharmane, and harmine) that could effectively ionize EGCG. The best solvent (MeOH, ACE, or ACN) for preparation of the EGCG was then evaluated. D-EGCG was also measured under the same condition of EGCG.

### Detection of EGCG spotted on a mouse liver section

EGCG (5 mM) was diluted with 80% MeOH or 80% ACE to a concentration of 100 μM. Ferulic acid, 1,5-DAN, harmane, norharmane, and harmine were used as matrix candidates in this experiment. Each matrix candidate was dissolved in a solvent as follows: (1) 1,5-DAN and ferulic acid were dissolved in 80% ACE (10 mg/mL), (2) harmane, norharmane, and harmine were dissolved in 80% MeOH (10 mg/mL). Slices (10 μm thick) of normal mouse liver were prepared with a cryostat and then thaw-mounted onto a SUS304 stainless steel slide. Ten microliters of EGCG (100 μM) was mixed with an equal volume of one matrix solution (10 mg/mL). The EGCG-matrix mixture (1 μL) was spotted onto a mouse liver section (50 pmol EGCG/spot, on-tissue, *n* = 3) and analyzed by MALDI-TOF-MS.

### MALDI-MSI of EGCG spotted on mouse tissues sections

Slices (10 μm thick) of normal mouse tissue (liver, kidney, or brain) were prepared with a cryostat and then thaw-mounted onto a SUS304 stainless steel slide. EGCG (5 mM) was diluted with 80% ACE to prepare a series of solutions (0, 2.5, 25, and 250 μM). Diluted EGCG solutions and a blank (0.2 μL, 80% ACE) were spotted onto the three mouse tissue sections (liver, kidney, and brain). The final amounts of EGCG at each spot were 0, 0.5, 5, and 50 pmol from the 0, 2.5, 25, and 250 μM solutions, respectively. The sample was allowed to dry for 5 min at room temperature before the matrix spray coating step. The matrix solution was prepared by dissolving 1,5-DAN in 80% ACE (10 mg/mL). The matrix was sprayed in a draft hood using an airbrush (Testor) at room temperature (22°C, 50% humidity). In the MSI experiment, MALDI-TOF-MS (AXIMA Performance) was used. Data were acquired in negative ionization mode with 50 μm spatial resolution (10 laser shots/data point), and the signals between *m*/*z* 100 and 1,000 were collected. Acquired MSI data were normalized with the intensity map of *m/z* 157, corresponding to the deprotonated 1,5-DAN peak, by using SIMedit software (Shimadzu), and obtained data were further processed with the freely available software BioMap software. The signal intensity of each imaging data in the figure is represented as the normalized intensity.

### EGCG treatment of mouse and tissue sample collection

After a 6-h fast, mice (six per group) were given a single dose of EGCG (2,000 mg/kg body weight, i.g.) as an aqueous solution with sterile saline, and then sacrificed 1 h after administration. D-EGCG was also treated under the same condition of EGCG. Mouse brain, liver, and kidney were collected and washed with an ice-cold 0.9% NaCl solution. These tissue samples were embedded in Tissue-Tek O.C.T. compounds and stored at −80°C until analysis by MALDI-MSI. A portion of each tissue was frozen directly and stored at −80°C for LC-MS.

### MALDI-MSI of EGCG and its metabolites in tissues sections from EGCG orally administrated mouse

EGCG-dosed mouse liver and kidney were sectioned (10 μm thick) with a cryostat and then thaw-mounted onto an SUS304 stainless steel slide. As a positive control, 250 μM EGCG (0.2 μL) was directly spotted on the tissue section. Then, the 1,5-DAN matrix solution (10 mg/mL in 80% ACE) was sprayed onto the tissue section. Imaging data were acquired in negative ionization mode with 50 μm spatial resolution (10 laser shots/data point), and the signals between *m/z* 100 and 1,000 were collected. The signal intensity of each imaging datum in the figure is represented as the normalized intensity. D-EGCG was also measured under the same condition of EGCG.

### LC-MS analysis of EGCG and its metabolites in tissue samples

Tissue samples were prepared and analyzed by Lambert's methods^23^ with modification. Tissue samples were homogenized in four volumes of ice-cold PBS (containing 2% (mass fraction of tissue mass) ascorbic acid) using a mechanical TOMY MSmash TM homogenizer (TOMY), and extracted with ethyl acetate. The ethyl acetate fractions were pooled and dried under N_2_ gas. Samples were reconstituted in 10% aqueous ACN. All tissue extracts were subjected to ESI-LC-MS analysis using a LCMS-IT-TOF instrument (Shimadzu). The instrument was fitted with a Luna 5u C18 (2) 100A column (250 × 1.0 mm, 5 μm, Phenomenex). The oven temperature was 40°C. The mobile phase was a binary gradient of solvent A (H_2_O containing 0.1% formic acid) and solvent B (MeOH). Solvent B was increased from 5% to 60% over 2 min, and then increased from 60% to 100% at 14 min. The mobile phase flow rate was 0.1 mL/min. Tissue homogenate extracts were resolved with sample buffer, 10% ACN with 10 μM 4-hydroxybenzophenone as an internal standard. Samples were filtered through a 0.2 μm polytetrafluoroethylene filter, and 6 μL was injected. The MS instrument was operated using an ESI source in negative ionization mode with survey scans acquired from *m/z* 100 to 800. Other EGCG derivatives were also measured under the same condition of EGCG.

### Isotopic fine structure analysis

Isotopic peaks of CHONPS elements quantitatively detected are closely matched to the natural abundance of each element. These data successfully lead us to determine the one elemental composition in a standard-independent manner^15^. Tissue section samples after each MSI experiment were subjected to ultrahigh-resolution MALDI-FT-ICR-MS analysis using a Bruker 7 tesla solariX™ FT-ICR mass spectrometer equipped with an ESI/MALDI dual ion source (Bruker Daltonk GmbH). The quadrupole and hexapole were equipped on the front of the ICR analyzer, and used as a mass isolator and ion storage, respectively. The collected ions were injected through a hexapole ion guide. The measurement was carried out with a Bruker Smartbeam-II laser, which was operated at a frequency of 500 Hz. Spectra were recorded on solariXcontrol software. Each mass spectrum was obtained from a single scan of 200 laser shots using 4 M data points in negative ion mode within a mass range of *m/z* 86–3,000. Mass resolution at *m/z* 457.07 (EGCG), 537.03 (EGCG-sulfate) or 633.10 (EGCG-glucuronide) was 150,000–250,000. The obtained data were processed with DataAnalysis software. EGCG and related metabolites were finally assigned by their isotopic fine structures.

### H&E staining

The tissues sections were fixed in 4% formaldehyde and stained with hematoxylin/eosin (H&E) according to standard protocol.

### Synthesis of D-EGCG and NMR

To a stirred solution of EGCG (30 mg), D_2_O (350 μL), acetone (70 μL), and deuterium trifluoroacetic acid (10 μL) were added at 0°C under argon. After stirring at room temperature for 1 h, the reaction mixture was concentrated by lyophilization. The residue was dissolved in H_2_O (1.0 mL). After stirring for 2 h at room temperature, the reaction mixture was concentrated by lyophilization to give EGCG-*d* (30.0 mg, quant.). EGCG was deuterated at the 2′ and 6′ positions in the B ring with 66% D. ^1^H-NMR (400 MHz, acetone-d_6_/D_2_O = 2/1) δ 6.92 (br-s, 2H), 6.56 (s, 2H,66% D), 5.94 (s, 1H), 5.93 (s, 1H), 5.33 (br-s, 1H, c), 4.93 (br-s, 1H, b), 2.90 (dd, 1H, *J* = 4.2, 17.4 Hz), 2.80 (d, *J* = 17.4 Hz, 1H).

## Author Contributions

Y.H.K., Y.F., T.H., M.S. and K.S. performed experiments. H.T. synthesized D-EGCG. D.Y., T.N., D.M., S.Y., H.W., K.Y., and H.T. were involved in the data analysis and interpretation. Y.F. and H.T. designed and supervised the project. Y.H.K., Y.F. and H.T. wrote the paper.

## Supplementary Material

Supplementary Information Supplementary Dataset
