# Supplementary material for: In situ label-free imaging for visualizing the biotransformation of a bioactive polyphenol
Source: Sci Rep. 2013 Sep 30;3:2805. doi: 10.1038/srep02805 (PMC3786292; doi:10.1038/srep02805)
Supplement: Supplementary Information — Supplementary Dataset [file srep02805-s1.doc]

**Supplementary information**

**Title: *In situ* label-free imaging for visualizing the biotransformation of a bioactive polyphenol**

Yoon Hee Kim^1,7^, Yoshinori Fujimura^2,7^, Takatoki Hagihara^1^, Masako Sasaki^1^, Daichi Yukihira^2^, Tatsuhiko Nagao^2^, Daisuke Miura^2^, Shinichi Yamaguchi^3^, Kazunori Saito^4^, Hiroshi Tanaka^5^, Hiroyuki Wariishi^1,2^, Koji Yamada^1^, and Hirofumi Tachibana^1,2,6,*^

^1^Faculty of Agriculture, Kyushu University, 6-10-1 Hakozaki, Higashi-ku, Fukuoka 812-8581, Japan. ^2^Innovation Center for Medical Redox Navigation, Kyushu University, 3-1-1 Maidashi, Higashi-ku, Fukuoka 812-8582, Japan. ^3^MS Business Unit, Life Science Business Department, Analytical and Measuring Instruments Division, Shimadzu Corporation, 1 Nishinokyo Kuwabaracho, Nakagyo-ku, Kyoto 604-8511, Japan. ^4^Bruker Daltonics K.K., 3-9 Noriya-cho, Kanagawa-ku, Yokohama 221-0022, Japan. ^5^Department of Applied Chemistry, Graduate School of Science and Engineering, Tokyo Institute of Technology, 2-12-1 Ookayama, Meguro, Tokyo 152-8552, Japan. ^6^Food Functional Design Research Center, Kyushu University, 6-10-1 Hakozaki, Higashi-ku, Fukuoka 812-8581, Japan. ^7^The authors contributed equally to this work. ^*^Corresponding author: Tel&Fax:+81-92-642-3008; E-mail address: tatibana@agr.kyushu-u.ac.jp

**Contents:**

**Supplementary Table S1**. Detection of EGCG corresponding to *m/z* 457 [M–H]^–^ and *m/z* 459 [M+H]^+^ by MALDI-MS using various matrix candidates.

**Supplementary Figure S1.** Structure of chemicals used for screening the matrix suitable for detection of EGCG.

**Supplementary Figure S2.** Evaluation of optimum conditions for detection of EGCG by MALDI-MS.

**Supplementary Figure S3.** Quantitative analysis of orally administrated EGCG in mouse tissues.

**Supplementary Figure S4.** NMR of the intact EGCG.

**Supplementary Figure S5.** NMR of D-EGCG.

**Supplementary Figure S6.** Analysis of D-EGCG by MALDI-MS and LC-MS.

**Supplementary Figure S7.** MALDI-MSI of EGCG-sulfate in EGCG-administrated mouse liver.

**Supplementary Figure S8.** MALDI-MSI of EGCG metabolites in EGCG-administrated mouse kidney.

**Supplementary Figure S9.** MALDI relative abundance of EGCG and its phase II metabolites within kidney tissue micro-regions.

**Supplementary Figure S10.** MALDI-MSI of EGCG and its metabolites on (a-d) liver and (e-h) kidney tissue sections from mouse after oral EGCG dosing (20 mg/kg).

**Supplementary Table S1. Detection of EGCG corresponding to *m/z* 457 [M–H]^–^ and *m/z* 459 [M+H]^+^ by MALDI-MS using various matrix candidates.**

| Chemicals | Intensity | | Chemicals | Intensity | |
| --- | --- | --- | --- | --- | --- |
|  | Negative | Positive |  | Negative | Positive |
| DHB | 58 | 0 | Acridine | 25,233 | 0 |
| CHCA | 0 | 0 | 9-AA | 16 | 0 |
| SA | 512 | 0 | 3,6-AD | 0 | 0 |
| HPA | 1,007 | 0 | 3-AQ | 537 | 0 |
| 5-ASA | 0 | 0 | 6-AQ | 5,072 | 0 |
| Salicylamide | 293 | 0 | 4-AP | 25 | 0 |
| DHAP | 262 | 0 | 4-NA | 62 | 0 |
| THAP | 9,716 | 0 | 1,5-DAN | 75,808,529 | 0 |
| HABA | 1,467 | 0 | DMAN | 0 | 0 |
| Caffeic acid | 10,581 | 0 | 2-NSA | 0 | 0 |
| Ferulic acid | 50,736,468 | 0 | 5-FU | 25 | 163 |
| Anthranilic acid | 0 | 0 | MTX | 1,831 | 0 |
| 2-AB | 208,205 | 0 | NFR | 0 | 0 |
| IAA | 216,431 | 0 | RB | 0 | 2,672 |
| Dithranol | 89 | 0 | Diamond | 0 | 0 |
| Norharmane | 48,008,145 | 0 | Silver | 0 | 0 |
| Harmane | 22,721,474 | 0 | Colloidal **graphite** | 0 | 0 |
| Harmol | 1,898 | 0 | TiO_2_ | 0 | 0 |
| Harmaline | 0 | 0 | SiO_2_TiO_2_ | 0 | 0 |
| Harmalol | 43,260 | 0 | BaTiO_3_StTiO_3_ | 0 | 0 |
| Harmine | 25,206,089 | 0 |  |  |  |

**
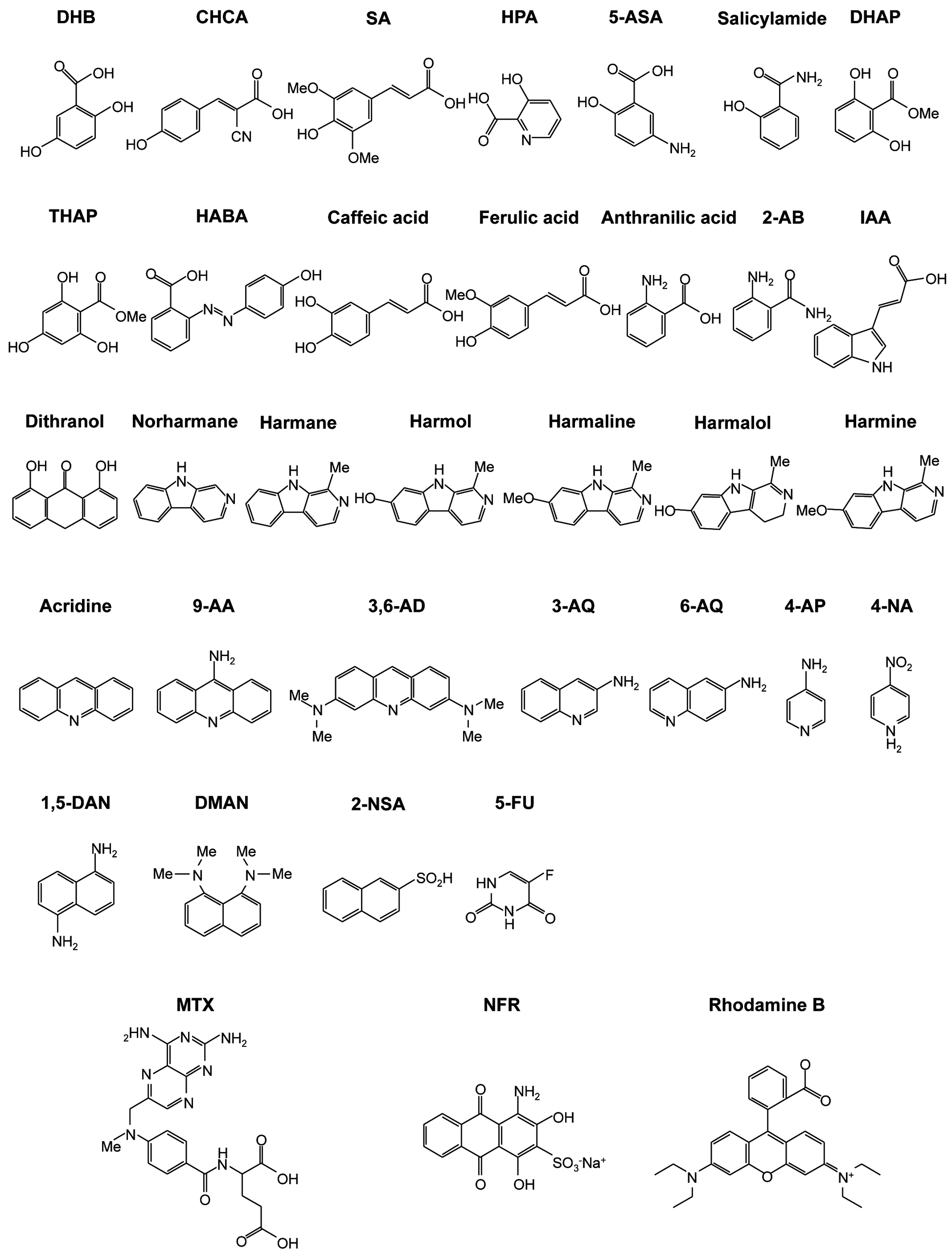
**

**Supplementary Figure S1.** **Structure of chemicals used for screening the matrix suitable for detection of EGCG.**


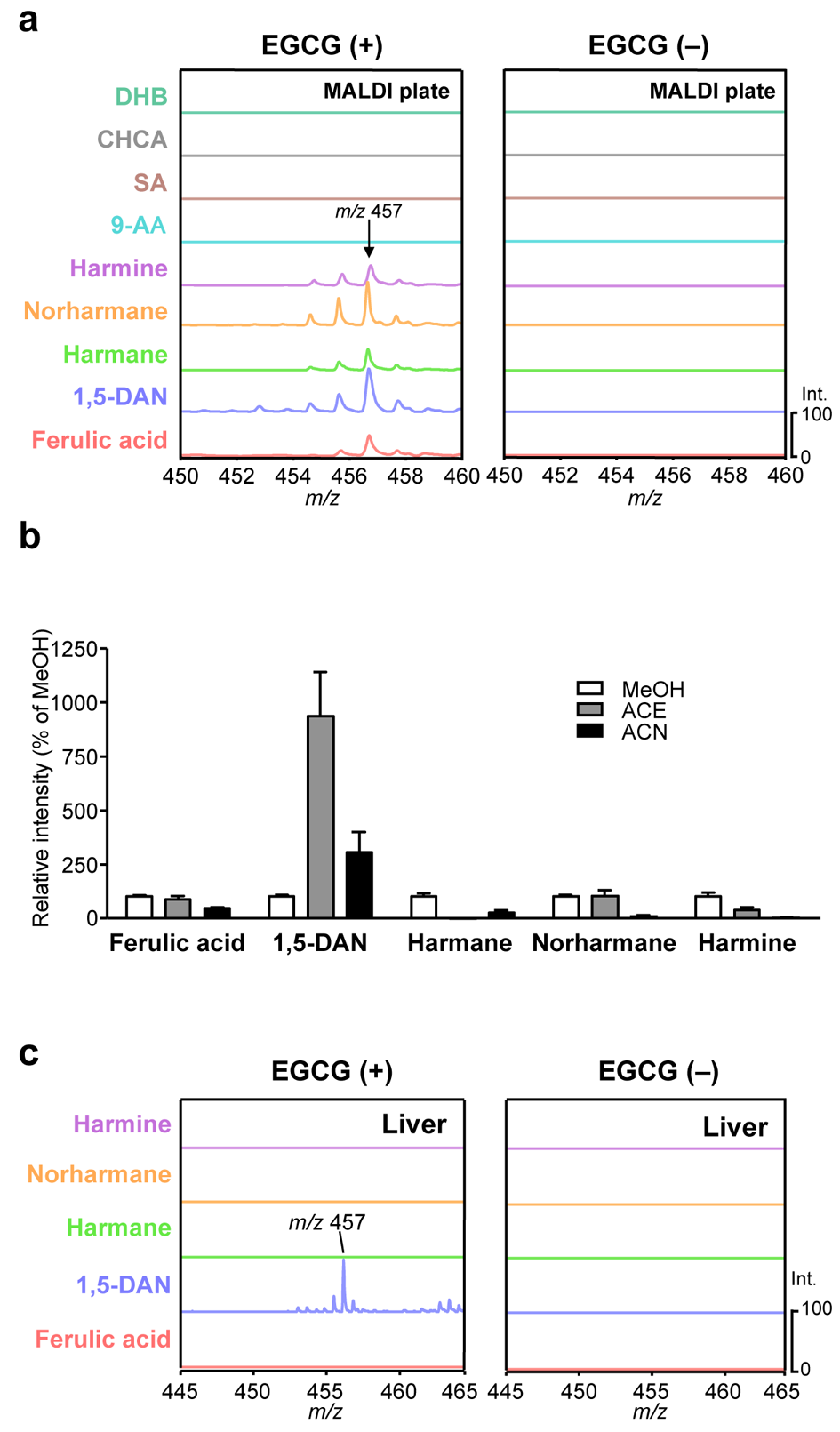


**Supplementary Figure S2. Evaluation of optimum conditions for detection of EGCG by MALDI-MS.** (a) EGCG and background peaks at *m/z* 457 regions on the stainless steel MALDI plate. MS spectral data were recorded in the presence or absence of EGCG (in 100% MeOH) in negative ionization mode. Each data was represented as the relative signal intensity when the intensity of the strongest intensity peak was 100 %. (b) Comparison of three representative solvents (100 % MeOH, 100 % ACE and 100 % ACN) for detection of EGCG in five matrix candidates on the MALDI plate. Bar graphs were represented as the relative signal intensity at the *m/z* 457 in negative ionization mode when the intensity of EGCG in MeOH was 100 %. Data was the mean±S.D. of three independent experiments (*n*=5 in each experiment). (c) Evaluation of EGCG and background peaks at *m/z* 457 on the liver section. MS spectral data were recorded in the presence or absence of EGCG (in 80% ACE) in negative ionization mode. Each data was represented as the relative signal intensity when the intensity of the strongest intensity peak was 100 %.


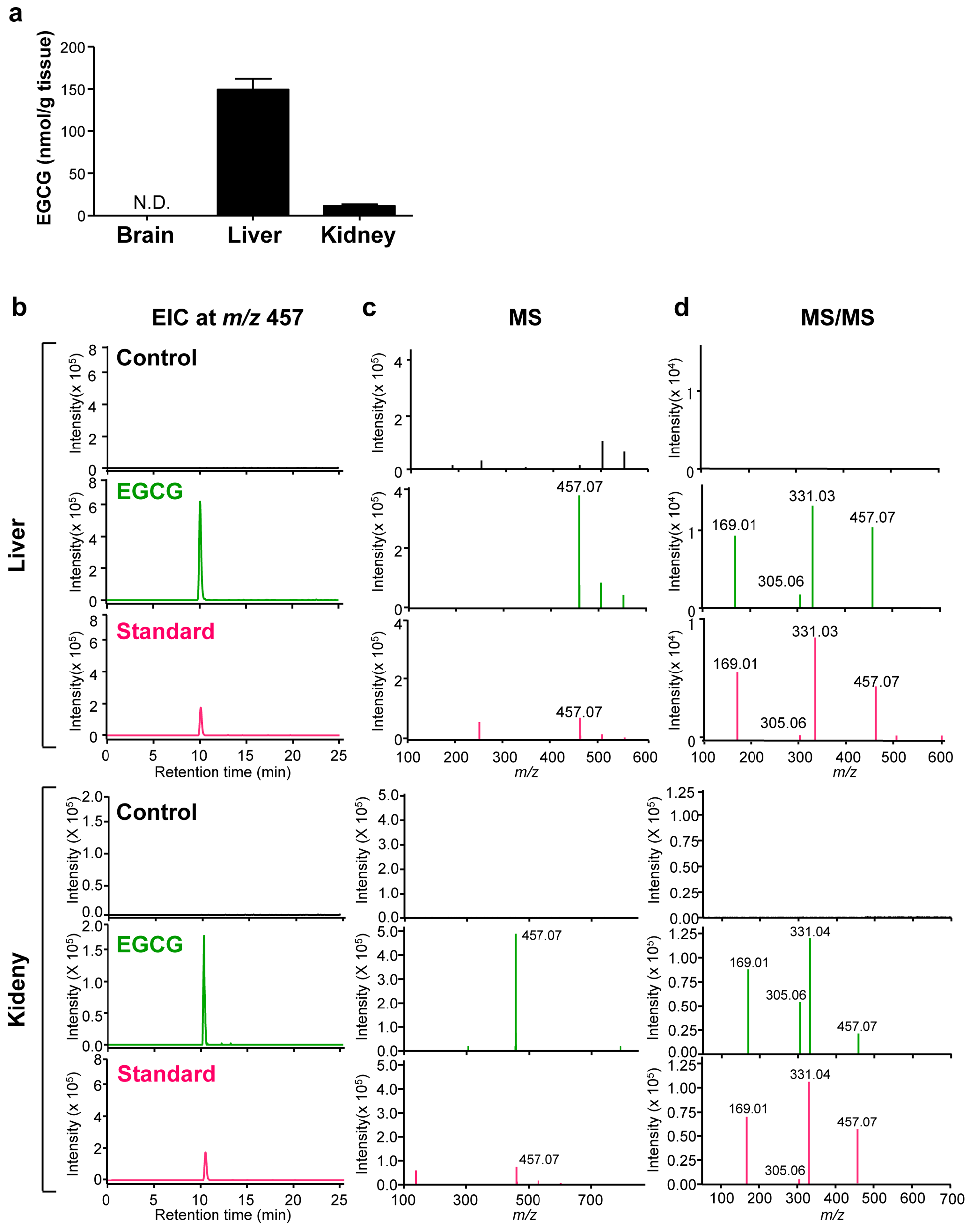


**Supplementary Figure S3. Quantitative analysis of orally administrated EGCG in mouse tissues.** (a) The amount of EGCG in each mouse tissue (brain, liver, and kidney) extract was determined by LC-MS analysis. The data are expressed as the mean±S.D. of six mice. Spectral data from liver and kidney are shown. (b) Extracted ion chromatogram (EIC) at *m/z* 457 of the EGCG standard (positive control) and liver extracts in both groups (Control and EGCG) in negative ionization mode. (c) Mass spectra and (d) tandem MS/MS spectra of EGCG at *m/z* 457 are also shown.


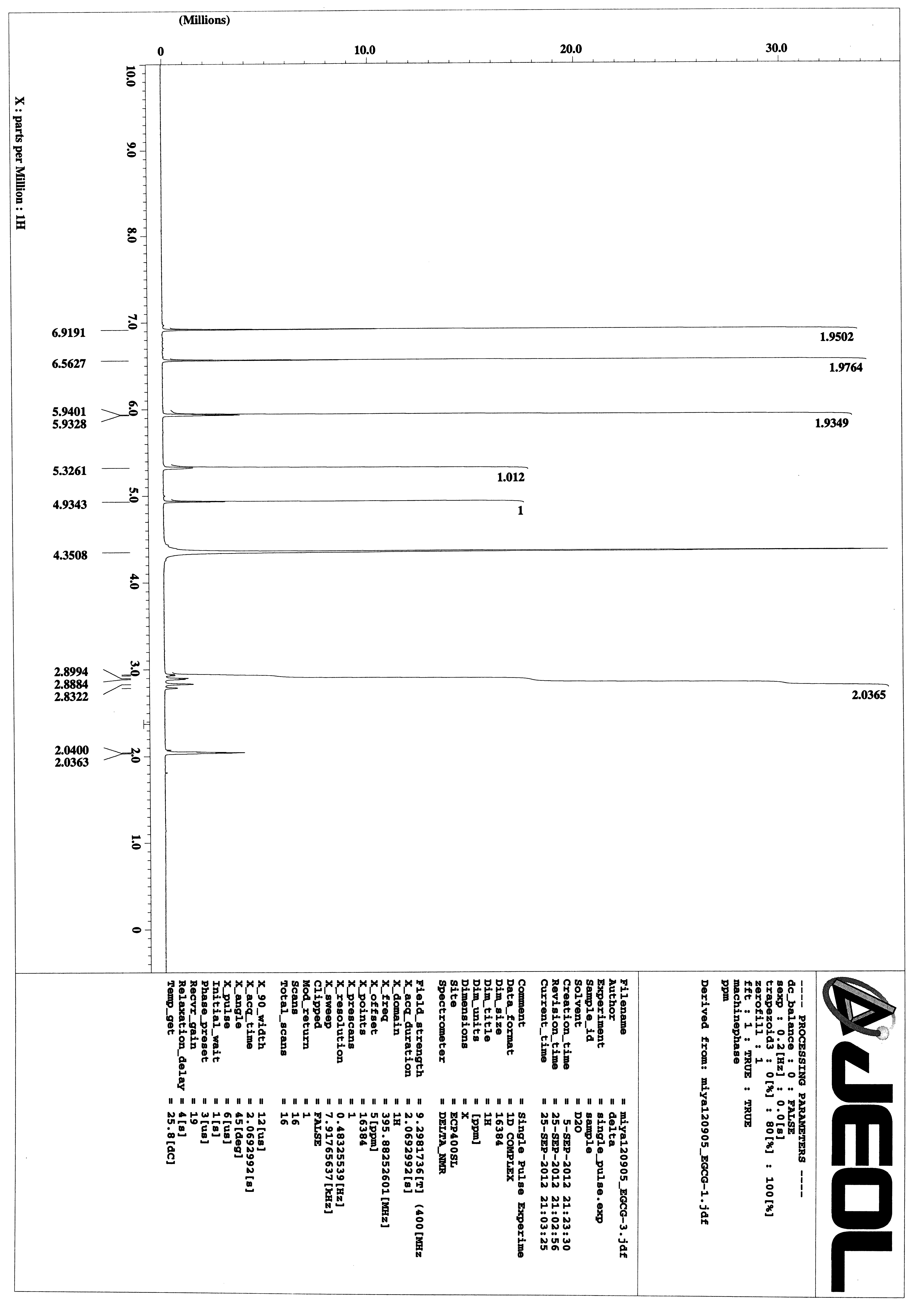


**Supplementary Figure S4. NMR of the intact EGCG**


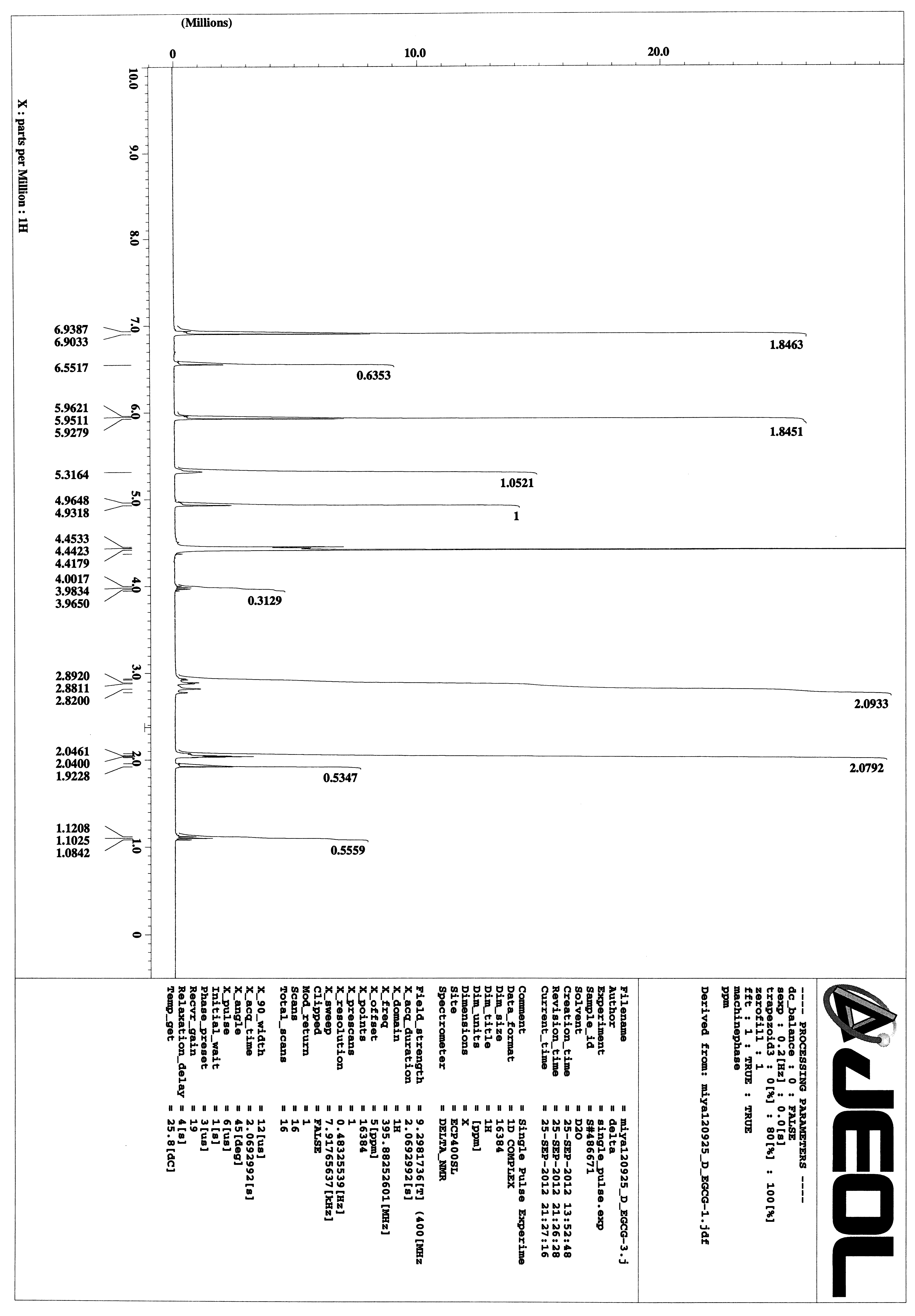


**Supplementary Figure S5. NMR of D-EGCG**


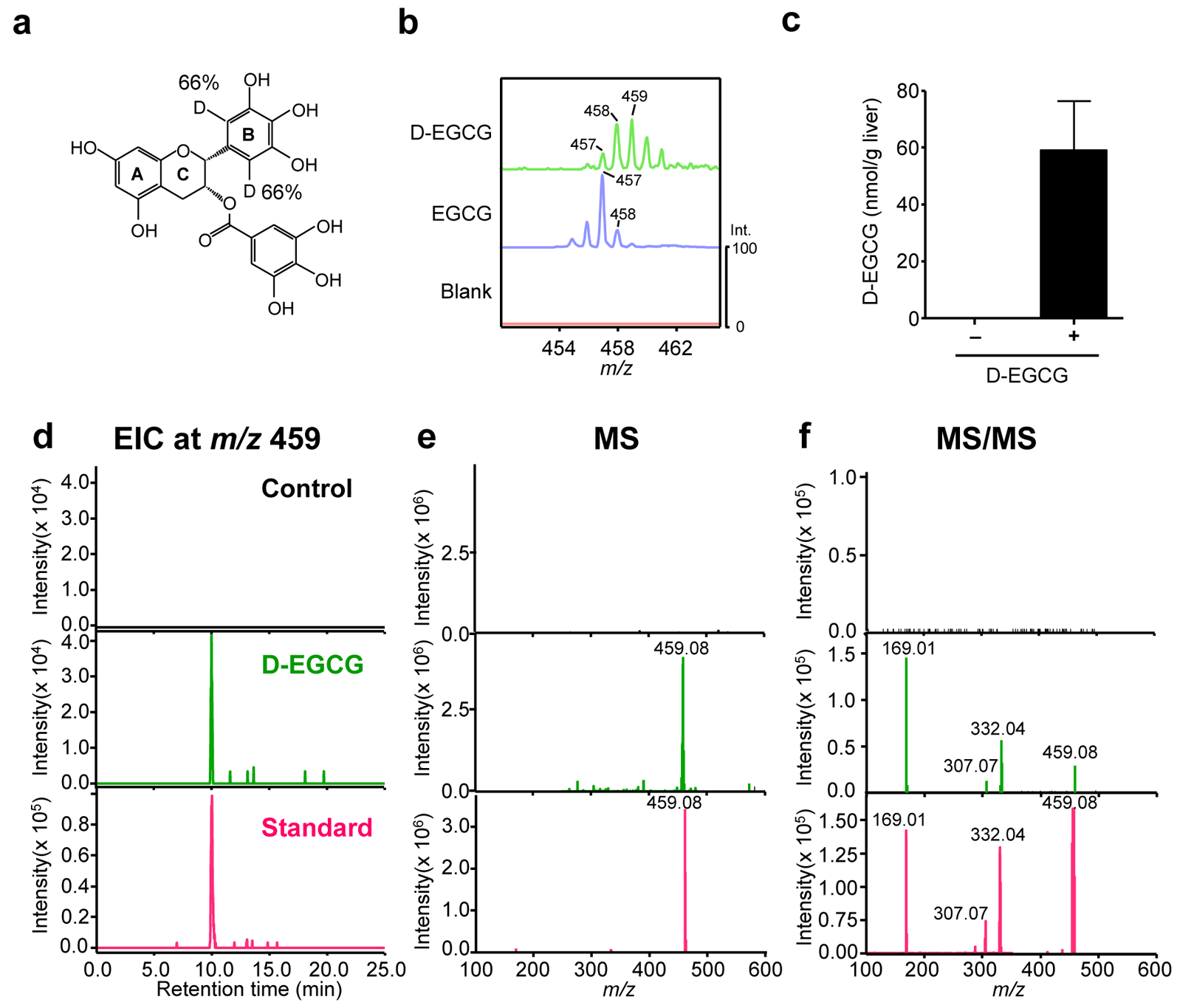


**Supplementary Figure S6.** **Analysis of D-EGCG by MALDI-MS and LC-MS.** (a) Chemical structure of deuterated EGCG (D-EGCG). (b) MALDI-MS spectra of D-EGCG on the MALDI plate in negative ionization mode. (c) Amount of orally administrated D-EGCG in mouse liver extract determined by LC-MS analysis. The data are expressed as the mean±S.D. of six mice. (d) EIC at *m/z* 459 of D-EGCG standard (positive control) and liver extracts in both groups (Control and D-EGCG) in negative ionization mode. (e) Mass spectra and (f) tandem MS/MS spectra of D-EGCG at *m/z* 459 are also shown.

**
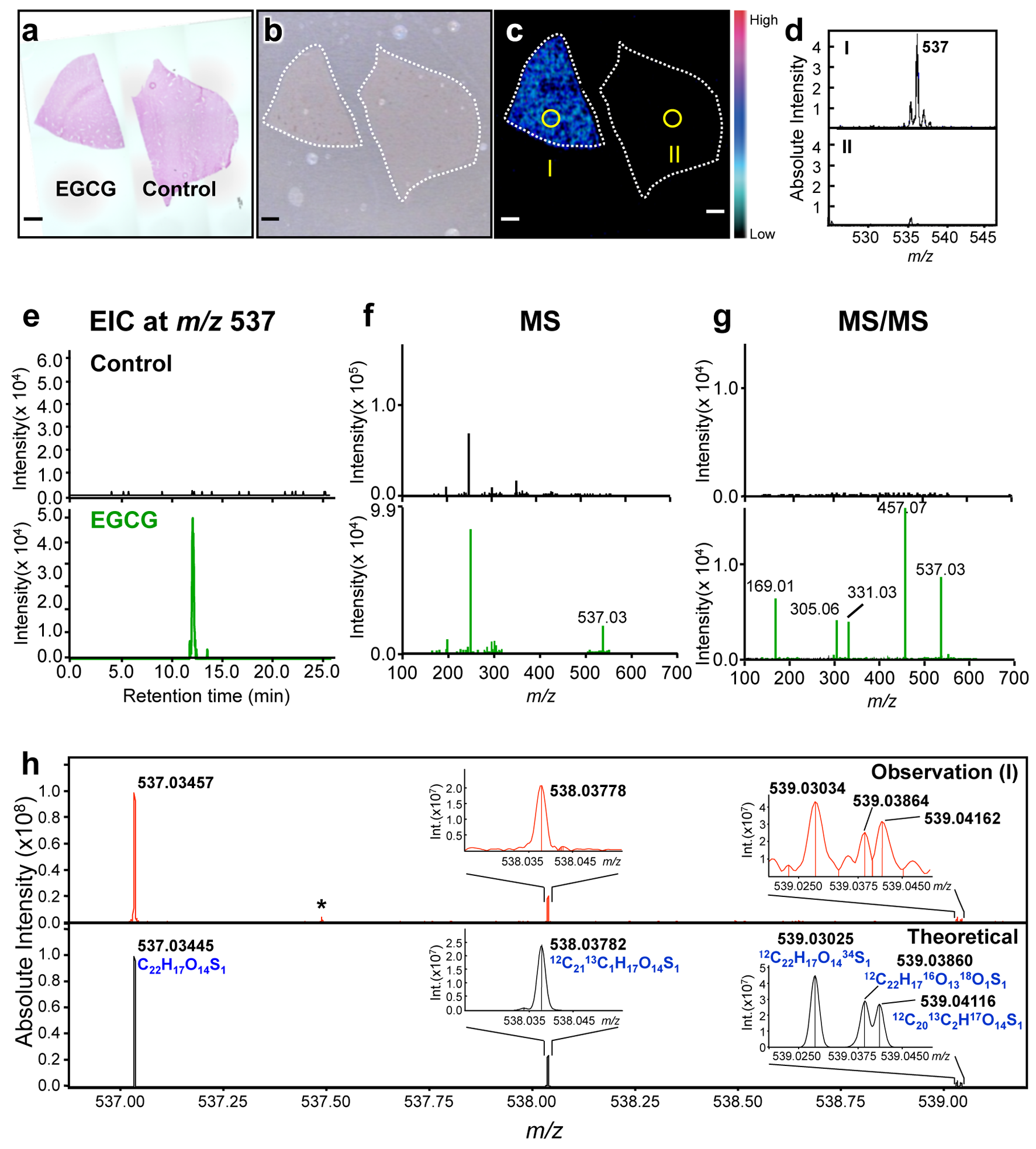
**

**Supplementary Figure S7.** **MALDI-MSI of EGCG-sulfate in EGCG-administrated mouse liver.** Three different images of (a) H&E staining, (b) optical microscopy, and (c) MALDI-TOF-MS at *m/z* 537, corresponding to the suggested EGCG metabolite peak [EGCG-sulfate (C_22_H_18_O_14_S_1_)–H^+^]^–^, in mouse liver section. MSI data were acquired with 50 μm spatial resolution with 10 shots/data point. Scale bar=1.0 mm. (d) Mass spectral observation of *m/z* 537 in the region of interest (I & II) indicated in panel *c*. (e–g) LC-MS analysis of the liver extracts is shown. (e) EIC at *m/z* 537 of liver extracts in both groups (Control and EGCG) at negative ionization mode. (f) Mass spectra and (g) tandem MS/MS spectra at *m/z* 537 are also shown. (h) Mass spectral observation of the suggested EGCG metabolite (C_22_H_18_O_14_S_1_) within the region of interest (I) indicated in panel c obtained using MALDI-FT-ICR-MS. Theoretical peaks of EGCG-sulfate (C_22_H_17_O_14_S_1_) were shown in negative ionization mode, and isotopic peaks were observed in the (M–H^+^+1)^–^ and (M–H^+^+2)^–^ regions. These peaks were theoretically assigned to the substitution of a stable isotope for each element. Asterisk shows background peaks.


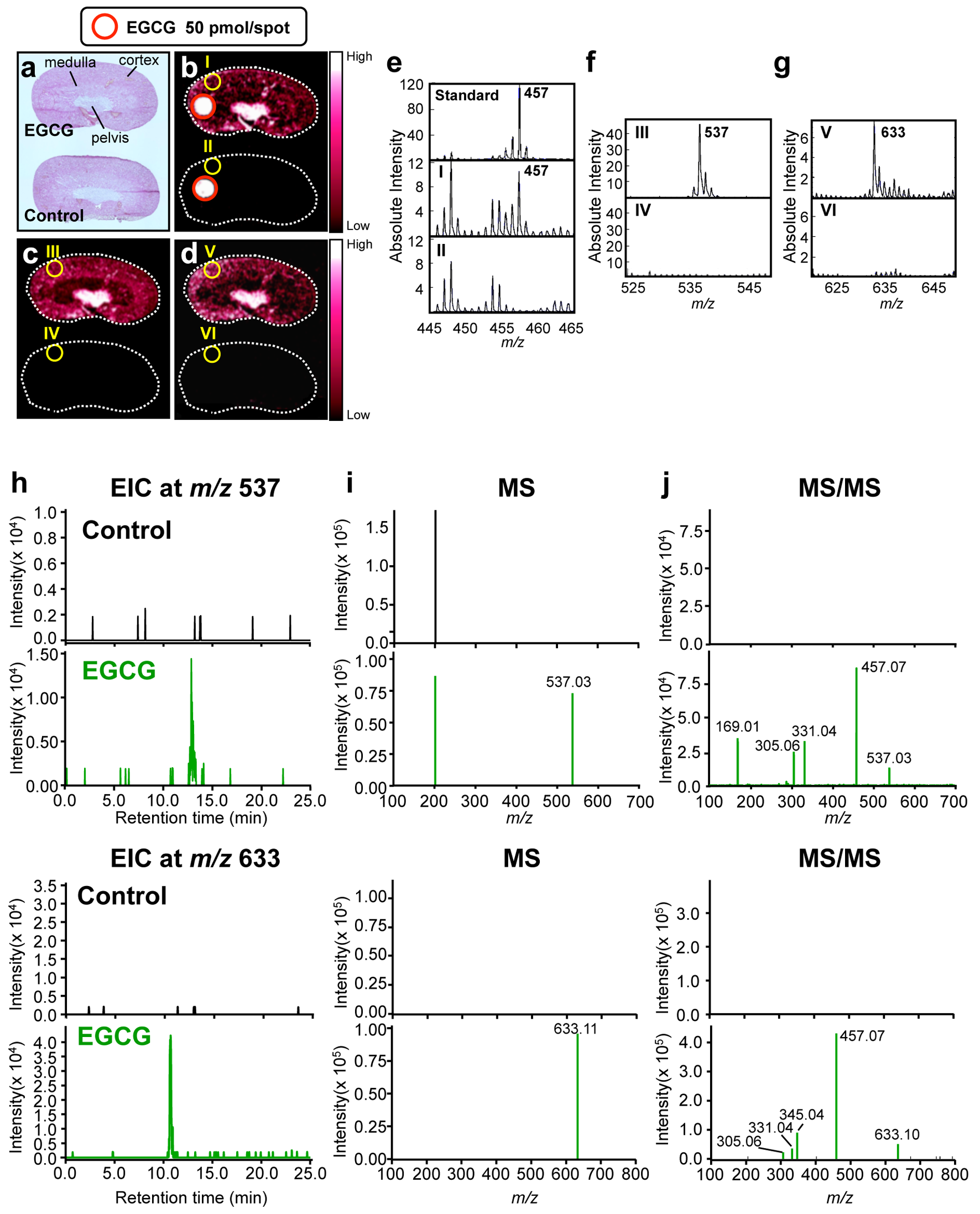


**Supplementary Figure S8.** **MALDI-MSI of EGCG metabolites in EGCG-administrated mouse kidney.** (a) H&E staining image of a kidney section. MALDI-MSI data corresponding to EGCG and its suggested metabolites, including (b) *m/z* 457 [EGCG (C_22_H_18_O_11_)–H^+^]^–^, (c) 537 [EGCG-sulfate (C_22_H_18_O_14_S_1_)–H^+^]^–^, and (d) *m/z* 633 [EGCG-glucuronide (C_28_H_26_O_17_)–H^+^]^–^. An additional EGCG spot (red circle) was visualized as a positive & internal control. MSI data were acquired with 50 μm spatial resolution with 10 shots/data point. Scale bar=1.0 mm. Mass spectral observations of (e) *m/z* 457, (f) 537, and (g) 633 were obtained within the region of interest (I & II, III & IV, and V & VI, respectively) indicated in panel b, c, or d. (h–j) LC-MS analysis of kidney extracts is shown. (h) EIC at *m/z* 537 or 633 of kidney extracts in both groups (Control and EGCG) in negative ionization mode. (i) Mass spectra and (j) tandem MS/MS spectra at *m/z* 537 or 633 are also shown.


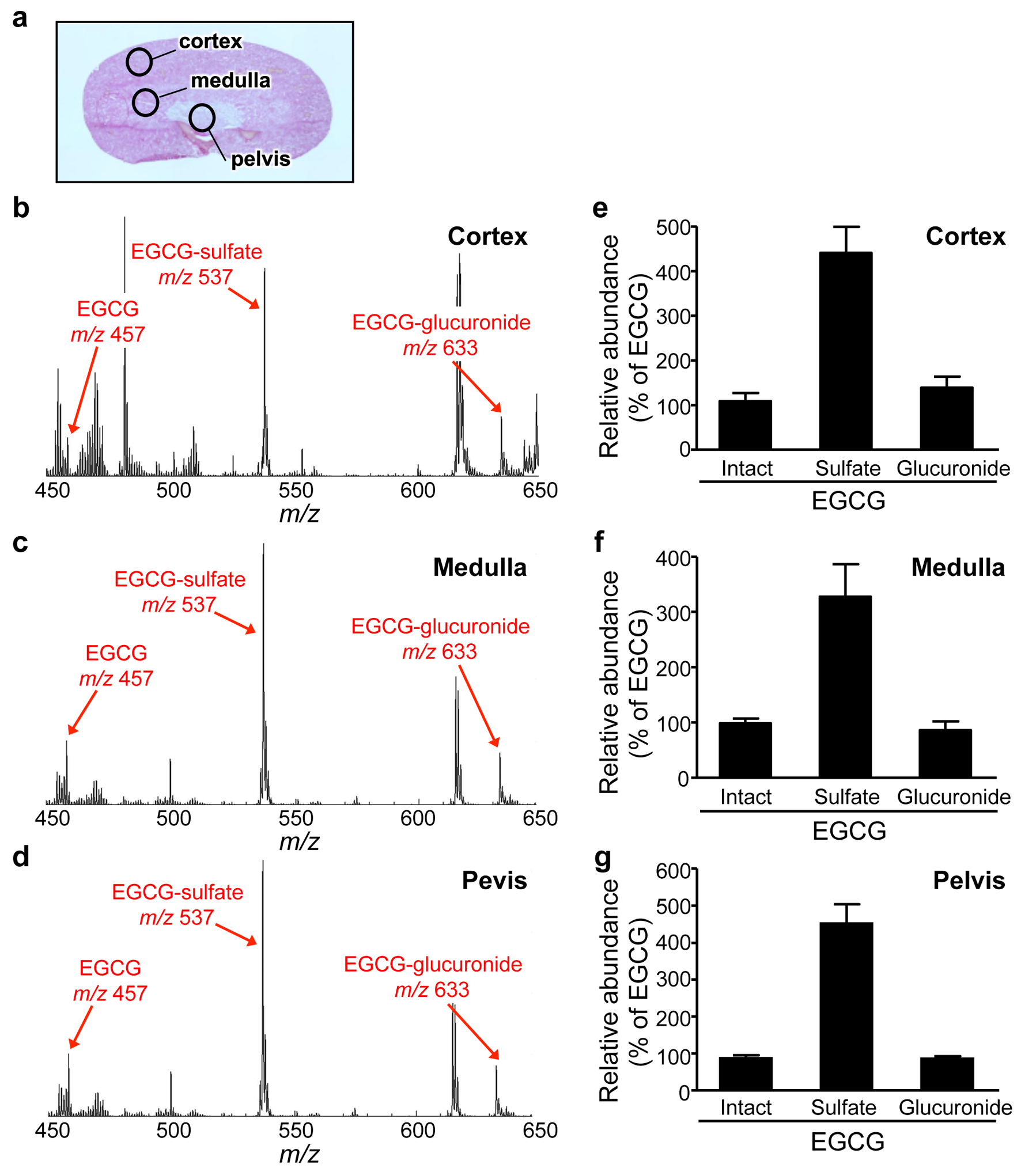


**Supplementary Figure S9. MALDI relative abundance of EGCG and its phase II metabolites within kidney tissue micro-regions.** (a) H&E staining image of a kidney section from EGCG-dosed mouse. (b–d) Average mass spectra of the region of interest indicated in panel a (cortex, medulla, and pelvis) were obtained by MALDI-TOF-MS. Each ion peak corresponding to EGCG, EGCG-sulfate, or EGCG-glucuronide was observed at *m/z* 457, 537, or 633, respectively. (e–g) In the three representative tissue micro-regions indicated in panel a, the relative peak abundance of EGCG phase II metabolites to EGCG is represented as the mean±S.D. of six mice.

**
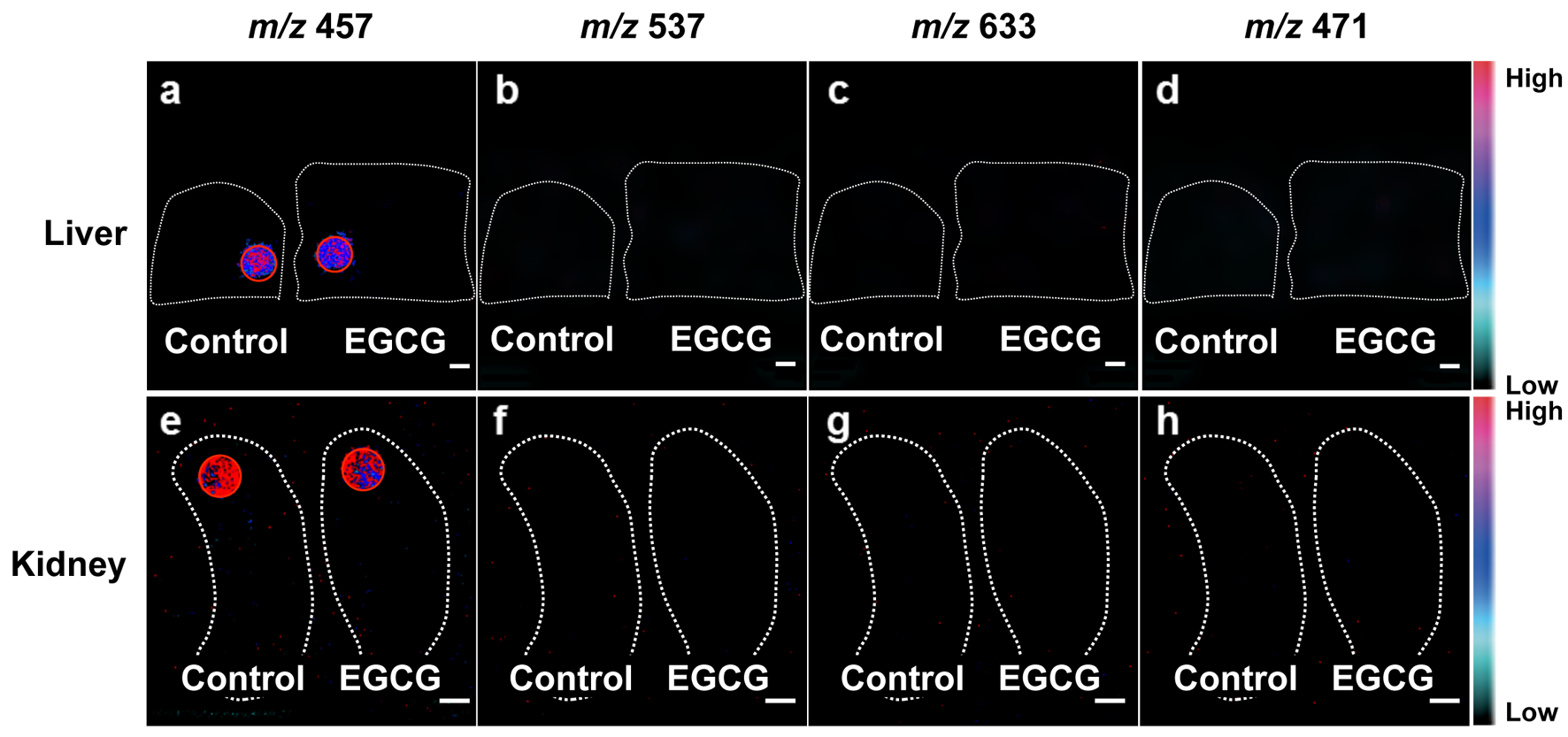
**

**Supplementary Figure S10. MALDI-MSI of EGCG and its metabolites on (a-d) liver and (e-h) kidney tissue sections from mouse after oral EGCG dosing (20 mg/kg).** Each different image is shown in (a, e) EGCG (*m/z* 457), (b, f) EGCG-sulfate (*m/z* 537), (c, g) EGCG-glucuronide (*m/z* 633), and (d, h) EGCG-methyl (*m/z* 471). An additional EGCG spot (red circle) was visualized as the positive and internal control.
